# Tourism and economic growth: A global study on Granger causality and wavelet coherence

**DOI:** 10.1371/journal.pone.0274386

**Published:** 2022-09-12

**Authors:** Chathuni Wijesekara, Chamath Tittagalla, Ashinsana Jayathilaka, Uvinya Ilukpotha, Ruwan Jayathilaka, Punmadara Jayasinghe

**Affiliations:** 1 SLIIT Business School, Sri Lanka Institute of Information Technology, Malabe, Sri Lanka; 2 Department of Information Management, SLIIT Business School, Sri Lanka Institute of Information Technology, Malabe, Sri Lanka; Newcastle University Business School, UNITED KINGDOM

## Abstract

This paper empirically investigates the relationship between tourism and economic growth by using a panel data cointegration test, Granger causality test and Wavelet coherence analysis at the global level. This analysis examines 105 nations utilising panel data from 2003 to 2020. The findings indicates that in most regions, tourism contributes significantly to economic growth and vice versa. Developing trade across most of the regions appears to be a major influencer in the study, as a bidirectional association exists between trade openness and economic growth. Additionally, all regions other than the American region showed a one-way association between gross capital formation and economic growth. Therefore, it is crucial to highlight that using initiatives to increase demand would advance tourism while also boosting the economy.

## Introduction

Tourism is one of the world’s major industries, and people have been travelling for pleasure since the dawn of time. It has become one of the fastest expanding sectors of the global economy in recent years. Tourism arose as a result of modernisation and contributed significantly to shaping the experience of modernity. Economic growth and tourism development are intertwined, according to previous literature, therefore, an increase in the general economy will support tourism development [1]. As a result, it’s critical to investigate how tourism and other factors (including macroeconomic) are linked to economic growth. Economic growth can be defined as an increase in the real gross domestic product (GDP) or GDP per capita. Global tourism, as a key contributory business, has contributed to approximately 10% of global GDP through possible employment opportunities, extending client markets, encouraging export trades, and gains from foreign exchanges [2, 3]. Another study that looked at the relationship between tourism and economic growth using variables like tourist receipts and tourism spending added to the literature by suggesting that tourism receipts impacted economic growth [4]. Additionally, according to Marin [5], tourism receipts have an upward link to the country’s economy and can thus aid in economic growth. Globally developed tourism business fosters economic growth over time, supporting the economy more than anticipated.

In recent years, research studies analysing the direction of the relationship between economic growth and tourism have been a popular area of interest in literature. A study of 12 Mediterranean nations in 2015 demonstrated a bidirectional causality relationship between tourism development and economic growth [6]. In a study conducted in Romania [7], a bidirectional causal relationship exists between GDP and the number of international tourist arrivals, whereas in an African study, a unidirectional causal relationship exists between international tourism earnings and real GDP, both in the short and long run [8]. According to previous research, this link appears to be both unidirectional and bidirectional.

Some of the processes by which tourism contributes to socioeconomic development include creating jobs, decreasing unemployment rates, and introducing of new tax income streams. In research conducted to investigate the relationship between tourism spending and economic growth in 49 nations, it was discovered that the two are inextricably linked, with a bidirectional causal relationship [9]. Investigating this relationship could be a useful for prioritising resource allocation across industries to improve overall tourism and economic outcomes.

Furthermore, a study based on 11 Asian regions discovers a close link between real international tourist revenues, capital formation, and GDP, confirming the tourism industry’s contribution to GDP [10]. Another study that looked at the relationship between tourism and economic growth based on tourist arrivals found that tourism is a good driver of economic growth [11]. This study looked into data of 94 countries, although there was no geographical examination of this association. Similarly, as previously mentioned, many authors have focused their research on a few countries or a single region when exploring the link between tourism and economic growth. The present study will contribute to filling the above-said research gap whilst providing an overall picture of the relationship between tourism and economic growth at the global level.

Many research papers have been written to determine the relationship between tourism demand and economic growth in diverse regions of the world. Based on certain regions, this link has been demonstrated to be bidirectional as well as unidirectional in the literature. The investigation of the relationship between tourism demand and global economic growth would provide a broad view of the relationship between these two factors. However, limited research has been done to examine this connection, which spans 18 years and includes regional data worldwide. Furthermore, because tourism is not the only element that influences GDP, other factors that considerably influence economic growth too must be considered. In the past, there hasn’t been much research conducted on the moderate impact of tourism on GDP. To address this gap in the literature, this research will examine the relationship between tourism demand and economic growth, as well as the moderating impact of variables such as gross capital formation and trade openness on economic growth in nations around the world. As a result, the current study focuses on all five regions, as there hasn’t been much research done on this topic.

The goal of this research paper is to examine the empirical relationship between tourism and economic growth along with the moderate impact of trade openness and gross capital formation for the worldwide regions. In four ways, the goals of this study can help improve the existing literature. Firstly, this study will be the most recent addition to the literature, focusing on an eighteen-year timeframe using panel data from 2003 to 2020. Secondly, this study will collect and analyse valid data from 105 countries including 42 countries in Europe, 25 countries in Asia & the Pacific, 18 countries in the Americas, and 20 countries from Africa and the Middle East region. The study’s emphasis on an 18-year time period and data from 105 countries allow the conclusions to be generalised and applied to any country. As a result, the study addresses one of the most significant flaws in the literature. Thirdly, in addition to the direct relationship between tourism on economic growth, this study attempts to examine the relationship between tourist receipts modulated by trade openness and gross capital formationon a region’s per capita GDP. These moderating effects on a country’s and region’s economic growth have yet to be investigated. Moreover, to the author’s knowledge, the wavelet technique hasn’t been used in previous research to analyse the relationship between per capita GDP and international tourist receipts. Additionally, analysis of this would produce precise and reliable data for future research and decision-making.

The next sections of the article are organised as follows: the first part analyses the existing literature, followed by the data used and the technique used in this investigation, then the findings and discussion, and lastly, the general conclusion of the study.

## Literature review

This section includes contributions to the literature by a variety of scholars from various nations and locations. The conclusions of the study done for a particular region were segregated into regions, whilst studies were divided according to the manner of causal relationship.

### Bidirectional causality between tourism and economic growth

The majority of earlier studies investigated the impact of tourism on economic growth in the European region. By adopting the Granger causality test Bilen, Yilanci [6] analysed the bidirectional causal connection between tourism development and economic growth, in the 12 Mediterranean countries with data from 1995-to 2012. Dritsakis [12] examined the impact of tourism on Greece’s economic development between 1960 and 2000, by using the Multivariate autoregressive and Granger causality tests. Here, the data revealed a ’Granger causal’ relationship between international tourism earnings and economic growth, a ’strong causal’ relationship between real exchange rate and economic growth, as well as simple ’causal’ relationships between economic growth and international tourism earnings, and real exchange rate and international tourism earnings. However, the above study conducted their research only for Greece. Further, the results of the above stated investigations based on 20^th^ century data, can vary with time. It is noteworthy that specially with the Eurozone crisis that started in 2009, Greece economy was among the severely affected in the region and hence, data do not reflect this situation. Surugiu and Surugiu [13] conducted a study using Romanian data, identified a long-term correlation between tourism development and economic growth.

According to the literature, several studies were conducted related to Tourism and economic growth. However, only a few studies have been conducted to analyse the causal relationship of both variables for countries worldwide. Most commonly utilised analytical tool is the Granger Causality test to identify the relationship between these two variables. A study conducted for 135 countries by Şak, Çağlayan [14] revealed that tourism revenue and GDP show bidirectional causality in Europe in contrast to unidirectional causality in America, Latin America, East Asia, South Asia, Oceania, Caribbean, and countries worldwide. However, the results of the above investigation were conducted based on data from 1995 to 2008, which can vary with time. Economic upheavals changes to economic policies in East Asia (including China, India) where geopolitical strategies are dominant, the impact of tourism revenue on GDP may not be significant. Moreover, Fahimi, Akadiri [15] tested the causality between tourism, economic growth, and investment in human capital in the microstates using data from 1995 to 2015. The results indicate that there is a bidirectional relationship between tourism and GDP. In the same period, Sokhanvar, Çiftçioğlu [16] performed a Granger causality analysis on 16 countries to investigate the causal relationship between tourism and economic development. The results proved bidirectional causality only in Chile. Further, this study found that seven countries do not show causality between variables. But as both studies were conducted only for selected countries, these results cannot be generalised about the global situation. Most recently, Pulido-Fernández and Cárdenas-García [17] explained the bidirectional link between tourism growth and economic development in 143 countries. According to them, tourism supports economic growth in the countries where tourism occurs. However, the study employed the level of economic development and tourism growth as a factor to cluster the countries for analysis; the results would most possibly change if another factor was used to cluster the countries.

### Unidirectional causality between tourism and economic growth

In the European region, a long-run link was tested between economic growth and tourism based on international tourist receipts, real GDP, and the real effective exchange rate for Croatian nations using quarterly data from 2000-to 2008. Using the Granger causality test as the analysis tool, the results proved that a positive unidirectional causal relationship exists between economic growth and foreign tourism revenues [18]. Moreover, by adopting the Granger causality test for the annual GDP, the number of foreign visitors to South Tyrol and the relative prices (RP) between South Tyrol and Germany from 1980 to 2006, Brida and Risso [19] proved that the causation from tourism and RP to real GDP is unidirectional. A study published in 2013 asserted the link between tourist spending and economic growth. For Cyprus, Latvia, and Slovakia, the study discovered a growth hypothesis. whereas a negative relationship for Czech Republic and Poland [20]. Furthermore, Lee and Brahmasrene [21] found that tourism has a positive impact on economic growth and is inversely related to carbon dioxide emissions, using the panel cointegration technique and Fixed Effect (FE) model for the European region. Besides, the majority of previous investigators employed the Granger causality test to determine whether a bidirectional or unidirectional link exists between tourism and economic growth among European regions.

For the Asian Region, Oh [22] conducted on the Korean economy revealed that there is a one-way causal relationship between economy-driven tourism growth by using the Granger causality test for the period from the first quarter of 1975 to the first quarter of 2001. Furthermore, according to the Granger causality test and co-integration, no co-integration exists between tourism and economic growth in the long run and Tourism-Led Growth Hypothesis (TLGH) did not exist in the short term. However, the author noted that in order to generalise the study’s findings, it is necessary to investigate the TLGH under economic conditions of numerous nations. Examining the most recent study in further detail, Wu, Wu [10] used a multivariate panel Granger causality test to show a growth hypothesis between real GDP and real international tourism receipt in China, Cambodia, and Malaysia. However, an opposite growth hypothesis has been validated in the Philippines, Hong Kong, Indonesia, and South Korea. In Macau and Singapore, an inverse growth theory has been discovered.

Many researchers have studied the relationship between tourism and the African continent’s economic growth, with various kinds of dimensions and methodologies. In the early 20s, Akinboade and Braimoh [8] used the Granger causality test to assert the link between international tourism and economic expansion in Southern Africa, where the findings demonstrated a one-way causal relationship between international tourism earnings to real GDP with the use of data from 1980 to 2005. Providing more evidence in the same period utilising the same method, Belloumi [23] too disclosed that tourism has a beneficial influence unidirectionally on economic growth. Moreover, Ahiawodzi [24] employed the Augmented Dickey-Fuller (ADF) test for unit root, cointegration test, and Granger Causality to investigate the cointegration and causality of tourism revenues and economic growth. It found a unidirectional causality from economic growth to tourism in Ghana as well as a positive relationship and cointegration in the long run. Similarly, Bouzahzah and El Menyari [25] also discovered significant unidirectional causation from economic growth to international tourist receipts in the long term by analysing data of Morocco and Tunisia. However, since these studies are limited to one or two countries in the region, researchers were unable to view the bigger picture as a region. The most recent study by Kyara, Rahman [26] was conducted based on data from Tanzania from 1989 to 2018, considering the country’s international tourist receipts, real GDP, and the real effective exchange rate as variables. Here, findings of Granger Causality, and the Wald test supported the existence of one-way causation between tourism and economic expansion.

Only a few researchers have studied the causation between tourism and economic growth in the Middle East region. Countries such as Bahrain, Saudi Arabia, and Jordan should implement strategies to boost tourist arrivals with receipts by uplifting their tourism to tourists from outside the Middle East region [27]. Also, the scholars conducted panel cointegration and causality test based on data from 1981 to 2008, which revealed that tourism has a long-term relationship with economic growth. However, this research might be improved to include additional countries in the region, allowing for a more realistic comparison. In the meantime, the impact of tourism on economic growth in oil-rich nations was stated by Alodadi and Benhin [28]. In Jordan, Kreishan [29] discovered a unidirectional causal relationship between tourism earnings and economic growth by investigating data from 39 years up to 2009 using the Granger causality test. The importance of tourism to economic growth was explained by Tang and Abosedra [30] using annual data for the period 1995–2010 in Lebanon. Their findings demonstrated that tourism and economic expansion in Lebanon have a long-term association as tourism and growth are cointegrated and the results supported that the Tourism led Growth hypothesis is valid in this country. However, this analysis was performed with a small sample without considering additional variables apart from tourist arrivals and the real GDP. Providing more evidence, the same conclusion was provided [31, 32], who tested data for Iran and Saudi Arabia, respectively. In addition, Ozcan and Maryam [33] claimed that measures to boost economic growth and development in the tourism sector of Qatar should be continued since a positive link exists between the said two factors. Ozcan and Maryam [33]. It may be determined from previous literature that the Middle East region exhibits a link between tourism and economic growth. Moreover, previous studies found that the tourism sector makes a small contribution to economic growth in oil-rich countries.

Many studies focusing on the countries of the American continent have deliberated the link between tourism and economic growth. According to Risso, Brida [34] the expenditure of international tourists has a favourable impact on Chile’s economic growth. The elasticity of real GDP to tourism spending (0.81) demonstrates that a 100% increase in tourism expenditure results in a long-run growth increase of more than 80%. With an elasticity of 0.35, the actual exchange rate also has a beneficial influence. This was examined using the Granger causality test as a basis for analysis using data from 1988 to 2009. Another study which was conducted by Brida and Risso [35], discovers that the causality of tourism and the real exchange rate to real GDP is unidirectional. Analysis of this study used the Granger test and the cointegrated vector model over data during the period 1988–2008. However, the above study only looked into data up to 2008. Similarly, Brida, Lanzilotta [36] analysed the causal relationship between Uruguay by adopting a Granger causality test. This study used variables such as GDP, Argentinean tourism expenditure, and the real exchange rate from 1987-to 2006, where it showed a positive relationship among the variables. However, this study was limited to Uruguay and Argentina. Using panel data from nine Caribbean nations from 1995 to 2007, a long-run relationship between economic growth and tourism was investigated by Payne and Mervar [18]. Here, researchers used international tourist arrivals per capita, real GDP per capita, and the real effective exchange rate. It proved that tourism has a large impact on per capita real GDP. Research conducted in Jamaica from 1970 to 2005 unveiled that increasing visitor receipts positively impacted on GDP. As a result, it was suggested that strategies should be focused on attracting more tourists, as this scenario would enhance not only tourism receipts but also Jamaica’s total economic growth [37]. However, the study described above, solely considered tourism receipts and GDP, excluding the other factors that affect GDP. Sánchez López [38] confirmed that international tourism has a positive influence on the Mexican economy by considering quarterly data from 1993 to 2017 and utilising GDP and tourist arrivals as variables.

Focusing on the worldwide studies, the case of Mediterranean countries, Tugcu [39] found a substantial and favourable correlation between tourism and economic growth. As these scholars affirmed, the relationship between economic growth and tourism has been studied for several groups of countries or nations. According to, the relationship between travel and economic growth varies per country, although European nations can experience economic growth through travel to European, Asian, and African nations. The most recent research, Enilov and Wang [40] examined the causal relationship between foreign tourist arrivals and economic growth using 23 developing and developed countries, in 1981–2017. It used a bootstrap mixed-frequency Granger causality approach using a rolling window technique to evaluate the approach’s stability and persistency over time concerning economic growth. The findings demonstrated that, in contrast to wealthy nations, the tourism industry in developing nations continues to be a major contributor in future economic growth.

In conclusion, many scholars have examined the connection between tourism and economic growth. However, the moderating impact of gross capital formation and trade openness with tourism receipts is yet to be studied. Moreover, limited studies were conducted to analyse the causal relationship between tourism and economic growth by employing the Granger Causality test. To fill this gap, this research investigates the direction of the causality between economic growth and demand for tourism whilst analysing the effect of gross capital formation and trade openness for the world regions.

### Conceptual framework

**Fig 1:**
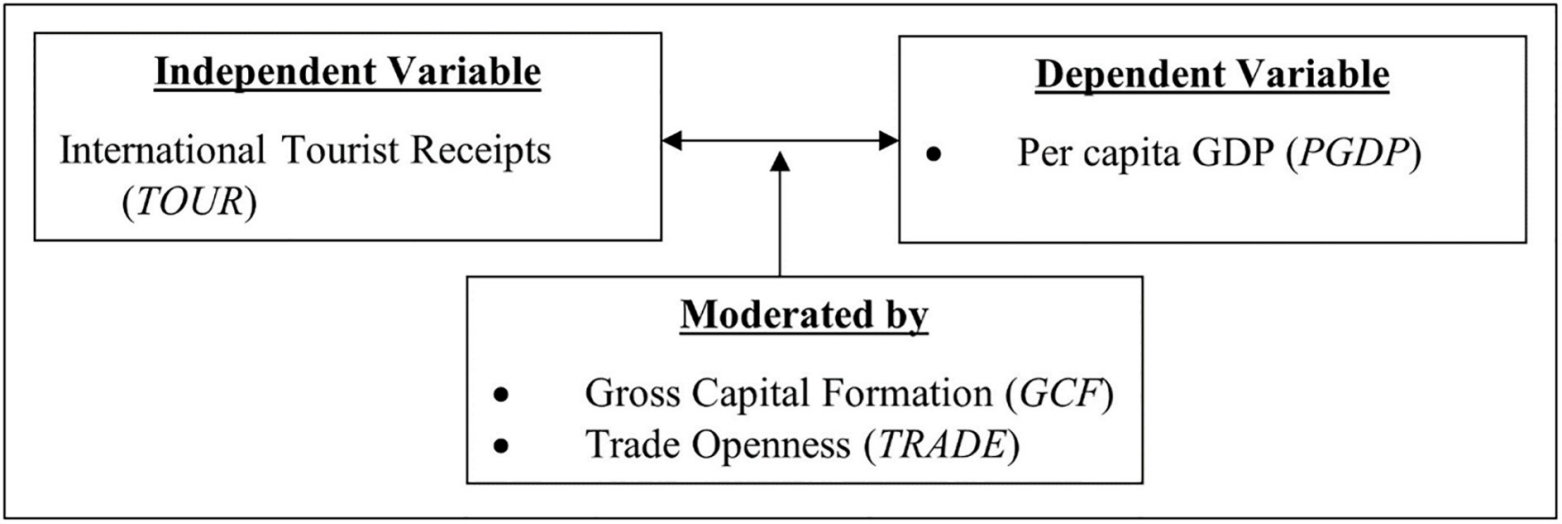
Conceptual framework. Source: Authors’ illustrations.

To address the gaps in this analysis, the conceptual framework was developed to investigate the relationship between tourism and economic growth, including the moderate effect of gross capital formation and trade openness, for worldwide regions as stated in the study’s objectives. Fig 1 depicts the conceptual framework for investigating the empirical relationship between tourism and economic growth, as well as the moderate influence of gross capital formation and trade openness, globally and for each region separately.

The endogenous growth theory, which often views economic growth as an endogenous product of an economic system rather than the result of factors that affect it from the outside, serves as the theoretical foundation [41]. In comparison to non-high-tech service industries like tourism, the endogenous growth theory tends to highlight the benefits of high-tech industries as possibly more favourable for high long-run growth. Yet, specialising in tourism can be strongly linked to higher returns, which in turn reinforces the benefits enjoyed by marketplaces, firms, and sectors.

## Data and methodology

This section presents a detailed view of the data, the statistical models employed in this study, and descriptive statistics for the variables.

### Data

This study was reviewed and approved by the SLIIT Business School and the SLIIT ethical review board. The following Table 1 illustrates the secondary data sources from which the information was gathered. The data file used for the study is presented in S2 Appendix.

**Table 1:** Data sources and definition of variables.

Variable	Definition	Measure	Source
*PGDP*	Per Capita Gross Domestic Product	(Current US$)	The World Bank
			https://data.worldbank.org/
*TOUR*	International Tourism Receipts	(Current US$)	UNWTO
			https://www.unwto.org/
			WorldData.info
			https://www.worlddata.info/
*GCF*	Gross Capital Formation	(% of GDP)	The World Bank
			https://data.worldbank.org/
*TRADE*	Trade Openness	(% of GDP)	The World Bank
			https://data.worldbank.org/

To measure economic growth across all regions, the current study employs yearly GDP per capita data from 2003. The amount of a country’s entire volume of goods and services produced relative to its total population is per capita GDP. To measure tourism growth, we use tourist receipts from 2003 until 2020. Tourism receipts were chosen over tourist arrivals because they incorporate both visitor arrivals and expenditure levels, resulting in a more accurate reflection of information on crucial aspects. Furthermore, the moderate impact on GDP per capita will be measured using gross capital formation and trade openness. Gross capital formation is a measure of a country’s yearly net capital accumulation as a proportion of GDP. The sum of goods and services and imports and exports represented as a percentage of GDP is known as trade openness. All the variables were converted as natural logarithms.

### Methodology

The causal link between *PGDP* and *TOUR* by analysing the moderating effect of *GCF* and *TRADE* is tested using the panel Granger causality test [42]. According to Wang, Zhang [43], to assess if the sequence of data is stationary the unit root test will be performed and the co-integration tests will be used to analyse the connection between the variables if they are non-stationary. Based on the co-integration test, the Panel Granger causality test will be adopted to determine the existence of the direction and the causal connection between tourism and economic growth by analysing the moderate effect of *GCF* and *TRADE*.

Thus, the following equation will be used to determine causality and its direction [44].

$$Y_{i,t}=\sum_{k=1}^{p}\beta_{i\;}Y_{i,t-k}+\sum_{k=0}^{p}{Ө}_{k\;}X_{i,t-k}+u_{i,t}$$ (1)

where, *Y* is the dependent variable (*i* and *t* denote the country and time, respectively) and *X* is the independent variable, *u*_*i*,*t*_ denotes the error term and *k* is the number of lags.

The CUSUM test was carried out to assess the stability of the parameters for countries in the regions separately. Brown, Durbin [45], Hawkins [46], Koshti [47] and Rasool, Maqbool [48] provided more explanation on how to identify and analyse the plot of CUSUM.

With the help of the above-mentioned equation and to prove the dynamics between the PGDP and *TOUR* from 2010 to 2020, the Wavelet Coherence approach is used in order to deeply analyse the existence of a correlation among the variables discussed. Goupillaud, Grossmann [49] developed the wavelet technique in its natural form, and the concept’s foundation is based on their expertise knowledge. A time series is decomposed into a frequency-time domain using the wavelet technique. Pal and Mitra [50], Adebayo and Beton Kalmaz [51], Kalmaz and Kirikkaleli [52] and Adebayo, Onyibor [53] explained how to analyse and the explanation of the wavelet coherence. The wavelet method is used in this study to further visually confirm the existence of a causal relationship among *PGDP* and *TOUR*.

The panel granger causality test was carried out using STATA whereas R Studio was used for the CUSUM test and Wavelet coherence.

## Empirical results and discussions

Before analysing Granger causality, Table 2 shows descriptive statistics for the major variables concerning worldwide countries and each region separately. This includes 1,890 total observations, of which 360, 324, 450, and 756 observations are for Africa & Middle East, America, Europe, and Asia & Pacific, respectively.

**Table 2:** Descriptive statistics of the key variables.

Countries		Variables			
		PGDP	TOUR (Millions)	TRADE	GCF
All Countries	Obs.	1,890	1,890	1,890	1,890
	Mean	17,408	10,100	92	24
	SD	21,433	21,300	61	7
	Min.	252	2	21	2
	Max.	123,514	242,000	443	69
Africa & Middle East	Obs.	360	360	360	360
	Mean	4,814	2,400	73	24
	SD	5,388	3,290	27	8
	Min.	254	4	21	2
	Max.	25,244	19,800	172	51
America	Obs.	324	324	324	324
	Mean	11,104	13,200	64	22
	SD	13,995	40,800	28	6
	Min.	908	81	22	11
	Max.	65,280	242,000	167	44
Europe	Obs.	450	450	450	450
	Mean	13,818	10,900	105	27
	SD	19,578	13,700	89	9
	Min.	252	2	21	7
	Max.	93,023	64,400	443	69
Asia & Pacific	Obs.	756	756	756	756
	Mean	28,245	11,900	106	24
	SD	24,615	16,400	56	6
	Min.	884	70	45	8
	Max.	123,514	81,700	380	58

Note: Obs., SD, Min. and Max. represent Observations, Standard Deviation, Minimum value, and Maximum value, respectively.

Source: Authors’ calculation based on data from the world bank, UNWTO, and WorldData.info.

**Fig 2:**
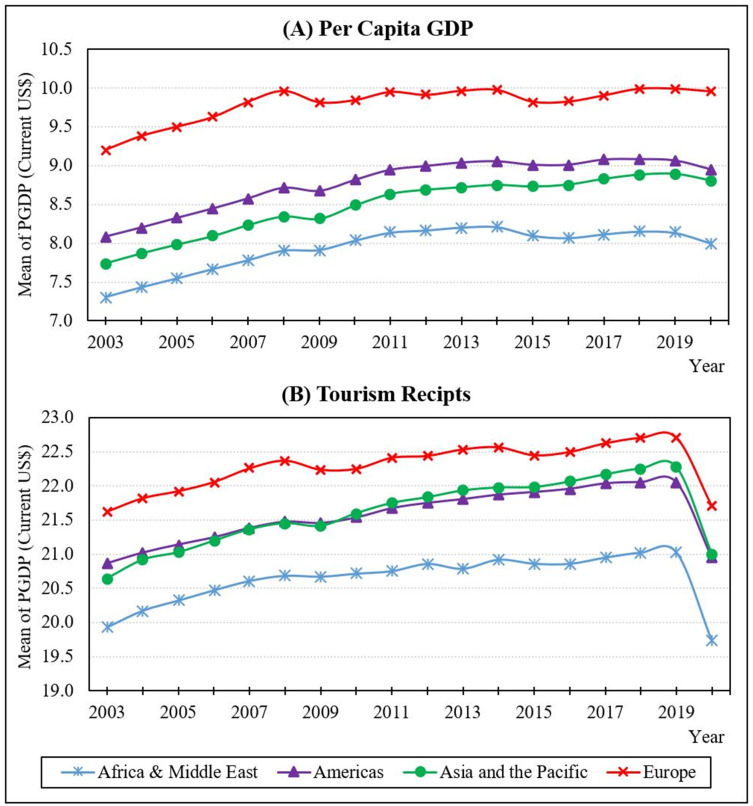
Region-wise means of per capita GDP and tourism receipts, 2003–2020. Note: The data points were converted as natural logarithms. Source: Authors’ illustration based on data from the world bank, UNWTO, and WorldData.info.

Fig 2 illustrates the mean *PGDP* and the mean *TOUR* for the world’s countries from 2003 to 2020, discovering the trend and patterns of key factors.

According to Fig 2(A), the African & the Middle East region has the lowest *PGDP* when compared to other regions, while the European regions have the highest *PGDP*. The *PGDP* of the Americas and Asia & Pacific areas fluctuated similarly until 2017, thereafter, the gap between these two countries narrowed. As indicated in Fig 2(B), the disparity in tourist receipts between America and the Asia-Pacific area has been nearly identical throughout the years. The European region has recorded the highest tourist receipts when compared to other regions. The graph shows that tourist revenues have dropped sharply after 2019. This is because tourism has been one of the most affected industries due to the covid pandemic. A massive drop in demand due to increased worldwide travel restrictions, including the closure of several borders worldwide led to tourism sector collapse.

The unit root tests are used in this study to determine if the data set of *PGDP*, *TOUR*, *TRADE*, and *GCF* is stationary or non-stationary. The following Table 3 shows the test results for unit roots.

**Table 3:** Testing for unit-roots.

Test	*ln(PGDP)*	*ln(TOUR)*	*ln(TRADE)*	*ln(GCF)*
**Levin-Lin-Chu unit-root test**	-14.6056***	1.0536	-3.7962***	-6.5461***
H_o_: Panels contain unit roots				
H_a_: Panels are stationary				
**Harris-Tzavalis unit-root test**	0.8178*	0.6890***	0.7783***	0.7175***
H_o_: Panels contain unit roots				
H_a_: Panels are stationary				
**Fisher-type unit-root test**	695.1157***	337.7582***	279.2116***	306.0028***
H_o_: All panels contain unit roots				
H_a_: At least one panel is stationary				
**Hadri LM test**	78.2386***	52.3402***	57.4555***	33.5307***
H_o_: All panels are stationary				
H_a_: Some panels contain unit roots				

Note: The symbols *, **, and *** represents 10%, 5%, and 1% significance level, respectively.

Source: Authors’ calculation based on data from the world bank, UNWTO, and WorldData.info.

The variables *PGDP*, *TRADE*, and *GCF* are stationary, according to the findings of the unit root tests. The Fisher-type unit-root test shows that some panels of the variable *TOUR* are stationary, but according to the Levin-Lin-Chu unit root test, the variable *TOUR* is nonstationary. As a result, the cointegration test is used to identify whether there is a long-term link between the variables *PGDP* and *TOUR*.

**Fig 3:**
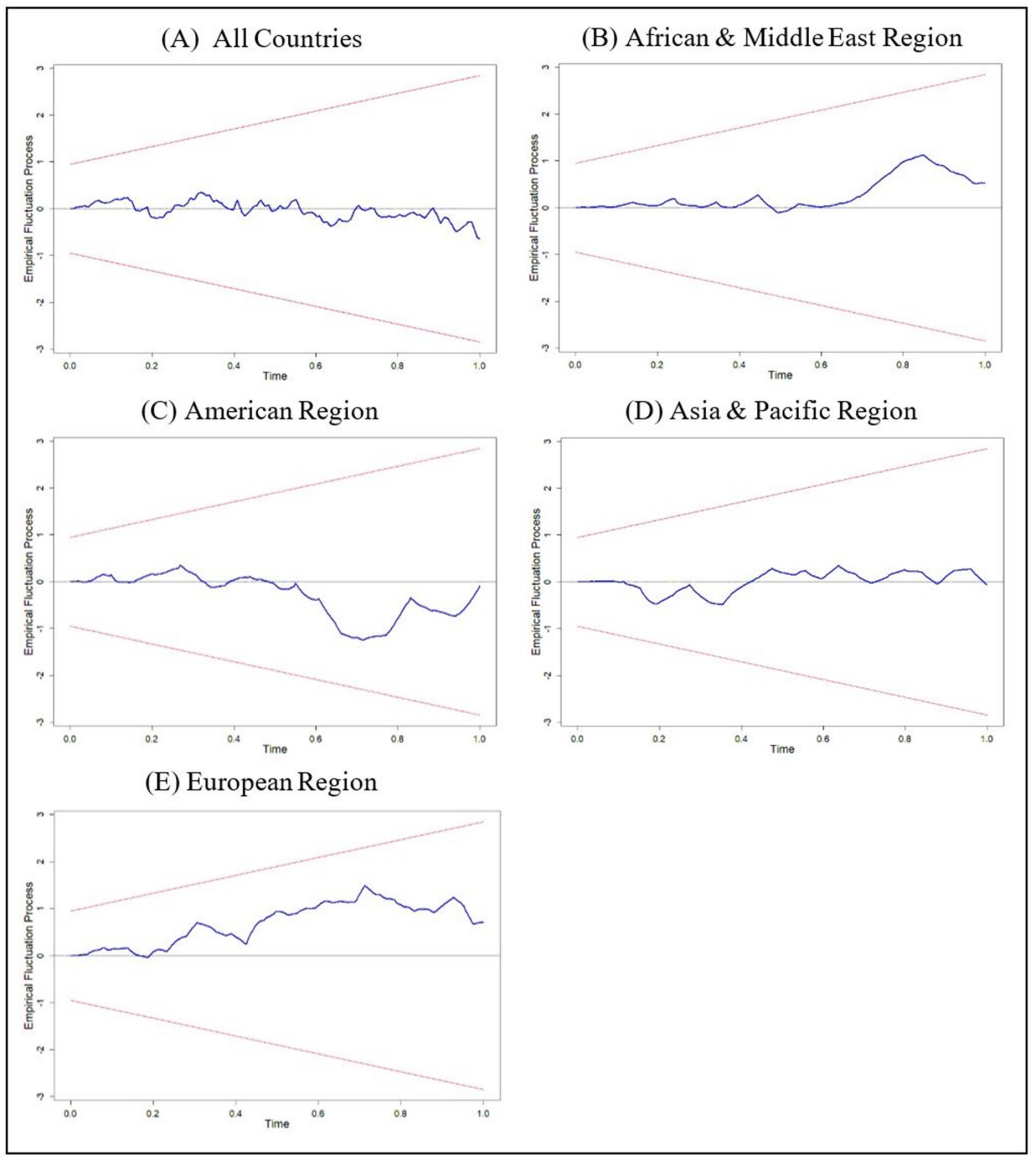
Recursive CUSUM plot for stability test. Source: Authors’ illustration using R-Software.

Table 4 presents the panel data cointegration test and results of the unit root tests proved that the variable *TOUR* is nonstationary. The findings of all the tests, except the Kao cointegration test, indicated that *PGDP* has a long-term connection with *TOUR*. It is possible to claim that there is at least a one-way Granger causality as the variables are co-integrated. According to the results of the stability test in Fig 3, the blue line in in the plot of recursive CUSUM does not cross the red line, it provides strong support that the model fits the data and that the variables are stable for all regions.

**Table 4:** Panel data cointegration test.

Panel Data Cointegration Test	*ln(PGDP) & ln(TOUR)*	Results
**Kao test**		The co-integration relationship exists (According to Modified Dickey-Fuller)
H_0_: No cointegration		
H_a_: All panels are cointegrated		
• Modified Dickey-Fuller t	-1.4976*	
• Dickey-Fuller t	-0.7745	
• Augmented Dickey-Fuller t	1.1361	
**Pedroni panel co-integration test**		Co-integration relationship exists
H_0_: No cointegration		
H_a_: All panels are cointegrated		
• Modified Phillips-Perron t	5.7244***	
• Phillips-Perron t	6.1026***	
• Augmented Dickey-Fuller t	8.2511***	
**Westerlund test**	3.2165***	Co-integration relationship exists
H_0_: No cointegration		
H_a_: All panels are cointegrated		

Note: The symbols *, **, and *** represents 10%, 5%, and 1% significance level, respectively.

Source: Authors’ calculation based on data from the world bank, UNWTO, and WorldData.info

According to Table 5, a bidirectional causal relationship exists between *PGDP* and *TOUR* for all the regions. However, the existence of a bidirectional relationship between *TRADE* and *PGDP* was discovered for all the regions except for the European region. On the other hand, a one-way causal connection (unidirectional) between *PGDP* and *GCF* was discovered for the American region, whereas all other regions proved the existence of a two-way relationship (bidirectional) between *PGDP* and *GCF*.

**Table 5:** Test results for granger causality.

Regions	*ln(PGDP) to ln(TOUR)*	*ln(TOUR) to ln(PGDP)*	*ln(PGDP) to ln(TRADE)*	*ln(TRADE) to ln(PGDP)*	*ln(PGDP) to ln(GCF)*	*ln(GCF) to ln(PGDP)*
All Countries	6.1325***	5.7114***	6.2647***	19.1149***	6.0097***	12.5575***
Africa & Middle East	2.9036 ***	2.6590***	6.9842***	9.9236***	5.6217***	4.7730***
America	3.4977***	4.2348***	3.2112***	13.2330***	-0.0361	2.9242***
Asia & Pacific	2.9006***	3.0982***	2.0690**	14.4490***	3.5075***	4.6961***
Europe	3.1651***	2.0331**	1.3872	3.5646***	2.9404***	11.0240***

Note: The symbols *, **, and *** represents 10%, 5%, and 1% significance level, respectively.

Source: Authors’ calculation based on data from the world bank, UNWTO, and WorldData.info.

Based on the findings of all countries, it can be observed that all the estimated z values of the variables *PGDP*, *TOUR*, *TRADE*, and *GCF* are significant at 0.001. Therefore, with the current estimators, it can be stated that in most countries worldwide, tourism growth Granger causes economic growth and vice versa. Subsequently, it could be assumed that tourism can drive economic growth in a majority of countries and economic growth can boost tourism growth. Fahimi, Akadiri [15] asserted that tourism to real GDP has a bidirectional causality relationship, where GDP Granger causes tourism and vice versa. However, Enilov and Wang [40] provide evidence for the validity of the economic-driven tourist growth in developing economies, while providing less support for developed ones. Similarly, according to Tugcu [39], Mediterranean area shows a favourable correlation between tourism and economic growth. This is likely attributable to a change in sample size, since our data set includes 105 nations and data spanning 12 years. But the research described above used a sample fewer than 25 nations. Furthermore, at the 1% significant level, the empirical findings prove that the *PGDP* Granger causes *TRADE*, *GCF*, and vice versa. Implications of these are that in most nations, the variables *TRADE* and *GCF* in *PGDP* have predictive ability amongst each other.

Similar to the worldwide countries, the values of the African and the Middle East region along with the Asia and Pacific region showed a significant relationship. At the 1% significance level, a Granger causal link between *PGDP* and tourist receipts was discovered, i.e., This means that tourism leads to economic growth and vice versa in the African and Middle East regions, as well as the countries in the Asia and the Pacific region. This finding was reconfirmed in a previous study conducted in Lebanon where it concluded that a bidirectional Granger causality exists between tourism and economic growth in the short run [30] in the Middle East Region. Similarly, these results were validated in South Africa by Odhiambo and Nyasha [54]. Moreover, for the Asian and Pacific region, Wang, Zhang [43] confirmed that there is a bidirectional Granger connection between China’s domestic tourism and economic growth. Additionally, using the Granger causality test, Mohapatra [4] proved the same results for the Asian and Pacific regions. According to the findings of these studies, the governments of these regions should promote practices and policies that would benefit the tourism industry and the economy, as tourism growth stimulates general growth in the economy and vice versa. Tourist revenues have surged across the Asia-Pacific region along with *PGDP*, as the region has evolved into a popular tourism destination for all sorts of diverse tourists. The rich biodiversity of several countries in the Asia and Pacific region has sparked the development of numerous sectors that have increased GDP, which in turn has had a substantial influence on tourism. A few countries in the Asia and Pacific area offer as much natural beauty, which makes them popular tourist destinations. The hospitality, infrastructure, convenient accommodation, and variety of attractions in these countries offer a solid basis for the Asia and Pacific region’s tourism industry. The proportion of international tourist arrivals in the African region is relatively low due to the region’s political unrest, yet tourism is one of Africa’s most promising industries concerning economic growth. The Middle Eastern nations are situated in the middle of important geographical locations. This aspect made it easier to establish global economic connections, which helped the economic growth of the countries over time. The Middle East led urbanisation and other development strategies that gave the region the required infrastructure and setting for the tourist destinations to begin providing of travel and tourism services. As a result, the Middle Eastern countries are increasingly opening their doors to tourists. Moreover, according to the finding, the null hypothesis of the Granger Causality test for the variables *PGDP* to *TRADE*, *TRADE* to *PGDP*, *PGDP* to *GCF* and *GCF* to *PGDP* can be rejected at a 1% significant level.

In contrast to countries worldwide, the American region revealed that a significant connection exists between *PGDP*, *TOUR*, and *TRADE*. Findings of this study affirmed that a one-way causal connection exists only from *GCF* to *PGDP* in the Americas region. These results mainly indicate that an increase in tourism could increase economic growth in the American region and vice versa. Several American countries, such as the United States and Canada, have a well-established tourism industry that contributes significantly to their GDP and, in turn, their highly developed economic systems encourage the development of infrastructure and tourist destinations. Governments are actively implementing regulations that intend to improve the economic, biological, and social advantages that tourist industry may offer, whilst lessening the challenges that occur when this expansion is unprepared and uncontrolled. Overall, tourist growth patterns in the Americas area are favourable. For the nations of the Americas region, in order to guarantee that their measures to improve tourism are conducted within the larger framework of local, regional, and country’s economic targets. Furthermore, to assist the shift to a green and low-emissions, additional initiatives are also being made to incorporate sustainability in tourism policy and industry regulations.

Considering the European region, a significant connection exists only among the variables *PGDP*, *TOUR*, and *GCF*. As a result, these findings show that tourist revenue and *PGDP* are mutually influenced. Furthermore, a significant link between *TRAD*E and *PGDP* was identified only in European region nations, demonstrating that *PGDP* does not cause TRADE, but *TRADE* has the predictive potential over *PGDP* at a 1% significance level. Europe is regarded as the overall dominant participant in the tourism industry, which fosters economic growth, due to the increasing affordability of travel for bigger groups of people. As tourism directly affects economic growth, it is possible to obtain economic growth in the European region by safeguarding the environment, preserving natural resources, generating jobs, enhancing cultural variety, and respecting cultural traditions. Authorities should focus on developing the tourist industry to obtain high economic growth, and to improve tourism, essential efforts should be taken to enhance economic growth. This is because bidirectional causation exists between tourism development and economic growth of the 12 Mediterranean countries [6].

The summary of Granger-causality analysis results for *PGDP*–*TOUR*, *PGDP*–*TRADE* and *PGDP*–*GCF* were presented in Table 6.

**Table 6:** Comparison of results among variables.

Regions	*PGDP—TOUR*	*PGDP—TRADE*	*PGDP—GCF*
All Countries	Bidirectional	Bidirectional	Bidirectional
Africa & Middle East	Bidirectional	Bidirectional	Bidirectional
America	Bidirectional	Bidirectional	One-way
Asia & Pacific	Bidirectional	Bidirectional	Bidirectional
Europe	Bidirectional	One-way	Bidirectional

Source: Authors’ illustration based on the test results generated.

All four regions show a bidirectional causal relationship between *PGDP* and *TOUR*. Furthermore, for the Africa & Middle East, America, Asia & Pacific areas, a two-way causal (bidirectional) link between *PGDP* and *TRADE* is demonstrated, whereas there is a one-way causal (unidirectional) link between *PGDP* and *TRADE* for the European area nations. When considering the causative relationship between *PGDP* and *GCF*, it is discovered that there is a bidirectional causal relationship in all regions except the Americas. In order to examine the relationship between variables among country’s separately, this study summarised the results of Granger Causality for the countries in each region separately in S1 Appendix.

Table 7 interprets the direction of the arrows and the frequency. The direction of the arrows will indicate whether the variables move in phase (rightward arrow indicating a positive correlation), or anti phase (leftward arrow indicating a negative correlation) and the cold (blue) regions of the figure indicates no correlation while the warm (red) regions depict the analysed variables are correlated. The wavelet coherence graph is identified according to the scale as the upper portion, middle portion, and lower portion which represents the short term, medium term and long term respectively.

**Table 7:** Interpretation of the wavelet coherence.

Direction of the arrows / Frequencies	Interpretation
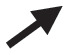	TOUR leads (cause) PGDP: In Phase
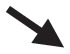	PGDP leads (cause) TOUR: In Phase
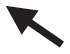	TOUR leads (cause) PGDP: Anti phase
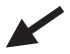	PGDP leads (cause) TOUR: Anti phase
0–2	Low frequency
2–6	Medium frequency
6–8	High frequency

Source: Authors’ illustrations.

**Fig 4:**
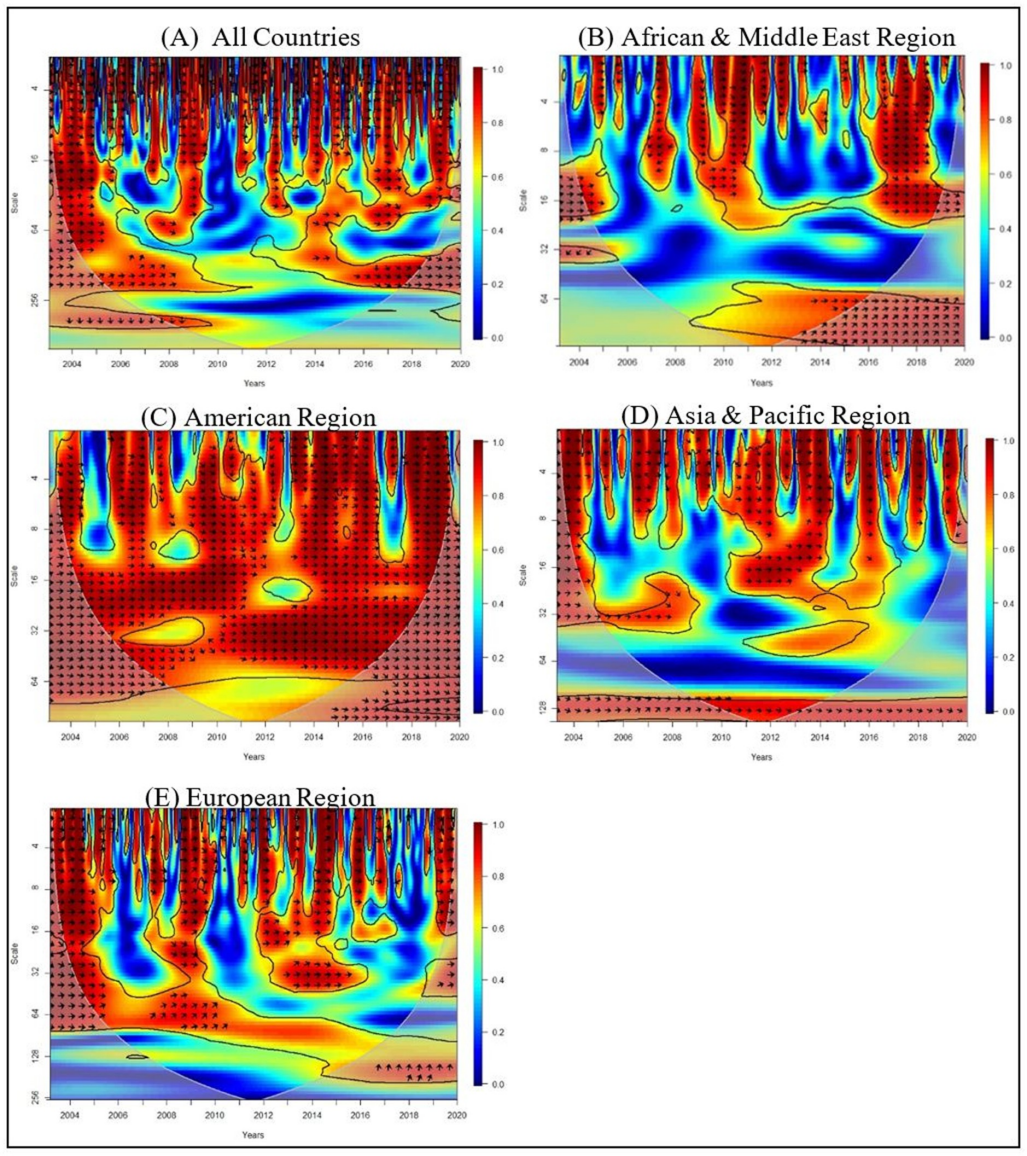
Wavelet coherence between *PGDP* and *TOUR*. Source: Authors’ compilation using R-Software.

The correlation between *PGDP* and *TOUR* for each region individually from 2003 to 2020 is shown in Fig 4. When considering the entire period, the arrows in Fig 4(A) are pointing right in the short and medium terms (high and medium frequencies), indicating a worldwide positive impact between *PGDP* and *TOUR* when assessing the entire period.

In Africa & Middle east region Fig 4(B) between 2009 and 2020, there are rightward arrows indicating a positive connection in the short and medium term with a high and medium frequency. Additionally, the rightward and downward arrows between 2009 to 2011 and 2016 to 2020 show that *PGDP* led *TOUR* in the short term with high frequencies. However, there is a negative association between 2006 to 2008 because of the existence of leftward arrows in the short with high frequency.

Overall, in American Region, Fig 4(C) demonstrates a favourable relationship with a high and medium frequency in all terms from 2003 to 2020. Furthermore, the rightward and downward arrows between 2008 to 2012 *PGDP* is leading to *TOUR*, in the short and long term (high and low frequencies).

Fig 4(D) illustrates a positive impact between Asia & Pacific Regions *PGDP* and *TOUR* in the short term with high and medium frequency over the years from 2003 to 2019, expect 2005 to 2006, 2008 to 2009, 2012 to 2013 and 2017 to 2018. There is a negative association in mentioned years because of the existence of leftward arrows in the short term with high frequency.

Fig 4(E) indicates a positive impact in the short and medium term (high and low frequencies) from 2003 to 2020 for the European region. Moreover, between 2006 and 2010, the arrows pointing right and up show a positive influence from *TOUR* to *PGDP* in the long term with low frequency. Similarly, the arrows in the medium term (medium frequency) between 2008 to 2011 and 2016 to 2018 are pointing downward and right, indicating that *PGDP* leads to *TOUR*.

Table 8 summarises the results of our Granger-causality analysis and wavelet coherence for *PGDP* and *TOUR*.

**Table 8:** Summary findings of Granger causality and wavelet coherence between PGDP and TOUR.

Regions	Granger Causality	Wavelet Coherence
All Countries	Bidirectional	Bidirectional
Africa & Middle East	Bidirectional	Bidirectional
America	Bidirectional	Bidirectional
Asia & Pacific	Bidirectional	Bidirectional
Europe	Bidirectional	Bidirectional

Source: Authors’ illustration based on the test results generated.

As the wavelet coherence technique captures the time dependence of the variables which is conjointly captured under the Granger causality approach, the findings revealed that overall finding of both techniques brings unanimous results, bringing justifications to the study. Both Granger Causality and Wavelet Coherence methods demonstrated that *PGDP* and *TOUR* had a bidirectional link in each region separately and globally. Where it demonstrates that tourism drove economic expansion and vice versa.

## Conclusion

This research was conducted to obtain evidence supporting the connection between tourism and global economic growth, using the panel Granger causality test with panel data from 2003 to 2020. The results of the link between *TOUR* and *PGDP* revealed a strong bidirectional connection. The results, firstly, indicated that tourism has the ability to boost economic growth in all regions, and vice versa. Secondly, a bidirectional relationship between *TRADE* and *PGDP* was observed in all regions except in the European region countries. Thirdly, the American area indicated a one-way causal association between *PGDP* and *GCF*, whereas the other regions revealed a two-way relationship between *PGDP* and *GCF*. Thus, based on these results, it is evident that tourism plays a substantial role in economic growth and vice versa across most regions. Therefore, it is important to emphasize that the use of demand-creation strategies to progress tourism would also boost economic growth.

Further to the bidirectional relationship between *TRADE* and *PGDP*, developing trade appears to be a powerful influencer in this study. Having said that, countries with increased tourism also have achieved developed trade and according to analysis, these two variables seem interrelated and mutually beneficial. It also suggests that in most countries, the variables *TRADE* and *GCF* in *PGDP* have the potential to forecast one another since the empirical findings show that the *PGDP* Granger causes *TRADE*, *GCF*, and vice versa. This paper differs from previous research in that it examines the relationship over 18 years, as well as the moderating impact of variables such as *GCF* and *TRADE* on economic growth in countries worldwide. Since the data set utilised in this study has a significant number of records, the analysis is more accurate, as the statistical soundness of results grows with the number of observations. As a result, the findings derived from this study could be generalised to the larger population including the entire world. In conclusion, it can be argued that tourism may be used as a catalyst for economic growth and vice versa. It is advised that nations in all regions proceed with caution when deploying more measures to attract visitors, as tourism has a strong influence *PGDP*. Moreover, the governments of these regions should support practices and policies that would benefit the tourism sector and eventually, the economy. The decision-makers should focus more effective tourism policies on addressing the demand generated by the rise in tourism-related businesses. Additionally, governments should promote investments in tourism-related industries to all types of investors as these ultimately boost the nation’s GDP. Global events such as the pandemic, economic downturns, and the war eruptions have triggered an unprecedented tourism economic crisis, due to the rapid and massive shock to the tourist industry. Due to this, tourism can be a vulnerable channel attracting refugees. This scenario can be risky as the increased pressure on the public finances exerts a higher burden on tax income and economic growth due to the migration of refuges in some countries. In this context, it is critical to overcome this predicament, as the negative repercussions could have a significant impact on the industry, and recovery will take time.

Here by examining the Wavelet Coherence graphs which had been drawn for the regions, American Region has the highest correlation between *PGDP* and *TOUR* from 2010 to 2017 compared to the other regions. Most of the graphs indicate a Bidirectional link, which is line with the findings of the panel granger causality. The visual representation of the bidirectional association between *TOUR* and *PGDP* in these results reflects the conclusions of the panel granger causality.

### Limitations

For this study, data were collected from 105 countries over 18 years, from 2003 to 2020. Other potential variables that influence tourism demand and economic growth, such as the real effective exchange rate, destination attractiveness, seasons, people’s spending capacity, security, urbanization, weather patterns etc., were not included in this study, which is a significant limitation. Moreover, the negative externalities of tourism and economic growth were not taken into account in this study due to the availability of data. For study purposes, countries were divided into regions, and those that depend heavily on tourism were not considered specific. As a result, the limitations mentioned above will need to be addressed in future studies. Future research studies should target to analyse the impact of tourism on economic growth and vice versa by adopting methodologies like the panel regression or generalised method of moments (GMM) which would further clarify the behaviour of these two variables more richly. Additionally, future study might assess the connection between tourism and economic development for each country in the relevant region independently.

## Supporting information

S1 Appendix Granger causality test results for the countries in each region. (DOCX)

S2 Appendix Data file. (XLS)
