# Supplementary material for: Tourism and economic growth: A global study on Granger causality and wavelet coherence
Source: PLoS One. 2022 Sep 12;17(9):e0274386. doi: 10.1371/journal.pone.0274386 (PMC9467361; doi:10.1371/journal.pone.0274386)
Supplement: S1 Appendix — (DOCX) [file pone.0274386.s001.docx]

**S1 Appendix.** **Granger Causality Test Results for the Countries in Each Region**

**S1.1 Appendix. Granger Causality Test Results - African and Middle East Region**

| **Regions** | ***ln(PGDP) to ln(TOUR)*** | ***ln(TOUR) to ln(PGDP****)* | **Summary**  ***PGDP - TOUR*** | ***ln(PGDP) to ln(TRADE)*** | ***ln(TRADE) to ln(PGDP****)* | **Summary**  ***PGDP - TRADE*** | ***ln(PGDP) to ln(GCF)*** | ***ln(GCF) to ln(PGDP)*** | **Summary**  ***PGDP - GCF*** |
| --- | --- | --- | --- | --- | --- | --- | --- | --- | --- |
| Africa and Middle East | 2.9036*** | 2.6590*** | **↔** | 6.9842*** | 9.9236*** | **↔** | 5.6217*** | 4.7730 *** | **↔** |
| Algeria | -0.0033 | 0.6098 | **⇼** | 1.3515 | 3.6488** | **←** | 0.0074 | 3.4950 *** | **←** |
| Angola | -0.2178 | 0.9860 | **⇼** | 1.8927* | 0.3236 | **→** | 0.4547 | -0.3907 | **⇼** |
| Botswana | -0.1549 | -0.2975 | **⇼** | -0.7002 | -0.5689 | **⇼** | -0.6970 | -0.4029 | **⇼** |
| Cameroon | -0.6969 | 0.8897 | **⇼** | -0.7041 | 0.7797 | **⇼** | 1.7616* | -0.6252 | **→** |
| Eswatini | -0.5481 | 7.2562*** | **←** | -0.5209 | 5.9817*** | **←** | -0.6751 | 8.2055*** | **←** |
| Ghana | 1.6446 | 0.2809 | **⇼** | 1.9554* | 0.2701 | **→** | -0.6948 | -0.7064 | **⇼** |
| Jordan | -0.4834 | 1.7399* | **←** | 1.6572* | 4.5317*** | **↔** | 0.9306 | 5.5308*** | **←** |
| Kenya | 0.8418 | 0.0121 | **⇼** | 0.5254 | 1.9307* | **←** | -0.7071 | -0.7047 | **⇼** |
| Lebanon | 6.1982*** | 1.8177* | **↔** | 3.1124** | 4.7690*** | **↔** | 28.1291*** | 3.8905*** | **↔** |
| Madagascar | 2.9553*** | -0.6348 | **→** | 4.6234*** | 1.5981*** | **↔** | -0.2102 | 0.1684 | **⇼** |
| Mauritius | -0.5861 | -0.6673 | **⇼** | 0.1148 | 2.1264** | **←** | -0.6980 | 1.6205 | **⇼** |
| Morocco | 2.1772** | -0.6101 | **→** | 0.7647 | -0.0277 | **⇼** | -0.6138 | 0.5769 | **⇼** |
| Namibia | 0.4502 | -0.6938 | **⇼** | 0.8162 | -0.5501 | **⇼** | -0.4772 | -0.7070 | **⇼** |
| Nigeria | -0.6132 | -0.1999 | **⇼** | 2.3495** | -0.3895 | **→** | -0.6926 | -0.6012 | **⇼** |
| Oman | 0.7256 | 0.6728 | **⇼** | 0.1703 | -0.5418 | **⇼** | -0.5216 | -0.3397 | **⇼** |
| Saudi Arabia | -0.6452 | -0.3382 | **⇼** | -0.7055 | 5.4155*** | **←** | -0.2361 | 1.5704 | **⇼** |
| South Africa | 1.7237 * | -0.6961 | **→** | 1.5323 | 3.0029** | **←** | -0.6885 | -0.6862 | **⇼** |
| Tanzania | 1.5752 | -0.4178 | **⇼** | 0.9997 | 9.1002*** | **←** | 0.0673 | -0.6530 | **⇼** |
| Uganda | -0.6618 | 2.4082** | **←** | 9.1915*** | 1.0778 | **→** | -0.2410 | 2.7101 | **⇼** |
| Zimbabwe | -0.6956 | -0.2264 | **⇼** | 2.8079** | 1.9017* | **↔** | 0.9435 | -0.6060 | **⇼** |

Note: The symbols *, **, and *** represents 10%, 5%, and 1% significance level, respectively. The symbols **↔, ⇼** represents bidirectional relationship and no significant relationship, respectively. Also, the symbols **←** and **→** represents one way causation and the arrow direction shows the direction of the causation.

Source: Authors’ calculation based on data from the world bank, UNWTO, and WorldData.info.

**S1.2 Appendix. Granger Causality Test Results – American Region**

| **Regions** | ***ln(PGDP) to ln(TOUR)*** | ***ln(TOUR) to ln(PGDP****)* | **Summary**  ***PGDP - TOUR*** | ***ln(PGDP) to ln(TRADE)*** | ***ln(TRADE) to ln(PGDP****)* | **Summary**  ***PGDP - TRADE*** | ***ln(PGDP) to ln(GCF)*** | ***ln(GCF) to ln(PGDP)*** | **Summary**  ***PGDP - GCF*** |
| --- | --- | --- | --- | --- | --- | --- | --- | --- | --- |
| America | 3.4977 *** | 4.2348*** | **↔** | 3.2112*** | 13.2330*** | **↔** | -0.0361 | 2.9242 *** | **←** |
| Argentina | -0.6615 | 0.0247 | **⇼** | -0.6311 | 1.8540* | **←** | -0.4509 | 3.5569*** | **←** |
| Bolivia | 0.1364 | -0.2565 | **⇼** | 1.1501 | 5.1186 *** | **←** | 0.1669 | 0.7896 | **⇼** |
| Brazil | -0.0291 | 0.3639 | **⇼** | 5.7019*** | -0.7071 | **→** | 3.5075*** | -0.0543 | **→** |
| Canada | -0.5109 | -0.5914 | **⇼** | 5.0635*** | 0.7065 | **→** | 0.2215 | -0.5410 | **⇼** |
| Chile | -0.6892 | -0.6918 | **⇼** | -0.4421 | 2.0699* | **←** | 0.1711 | 0.8531 | **⇼** |
| Colombia | 0.2863 | 0.6247 | **⇼** | -0.2425 | -0.6896 | **⇼** | -0.7070 | -0.4119 | **⇼** |
| Costa Rica | 2.3472** | 1.2640 | **→** | 0.3474 | 3.4623*** | **←** | -0.7068 | 2.1530** |  |
| Ecuador | 3.5548*** | 1.5044 | **→** | 0.4008 | 3.1626*** | **←** | 1.0943 | 0.1068 | **⇼** |
| El Salvador | 0.5320 | 0.4963 | **⇼** | -0.5647 | -0.6360 | **⇼** | -0.2882 | 0.5037 | **⇼** |
| Guatemala | -0.6685 | 0.5265 | **⇼** | -0.7054 | 5.5556*** | **←** | -0.7057 | 2.5696** | **←** |
| Honduras | 1.1468 | 5.3988*** | **→** | -0.7071 | 6.0623*** | **←** | -0.6678 | 3.5075*** | **←** |
| Mexico | 0.0379 | -0.6991 | **⇼** | -0.6968 | -0.5124 | **⇼** | -0.0949 | -0.4519 | **⇼** |
| Nicaragua | 1.3955 | 0.4030 | **⇼** | 2.3474** | 0.6099 | **→** | -0.1465 | 0.7357 |  |
| Panama | 5.4367*** | 1.4334 | **→** | 3.3671*** | 7.8200*** | **↔** | 2.8501*** | -0.4164 | **→** |
| Paraguay | -0.6601 | -0.0215** | **←** | -0.4870 | 4.6992*** | **←** | -0.5150 | -0.4675 | **⇼** |
| Peru | -0.0397 | -0.0215 | **⇼** | 0.3866 | 3.4054*** | **←** | -0.7047 | -0.6995 | **⇼** |
| United States | 0.1046 | 5.7680*** | **←** | -0.1422 | 1.1794 | **⇼** | -0.7059 | -0.5515 | **⇼** |
| Uruguay | 3.1203*** | -0.1185 | **→** | -0.5217 | 12.9824*** | **←** | 0.7950 | 1.2244 | **⇼** |

Note: The symbols *, **, and *** represents 10%, 5%, and 1% significance level, respectively. The symbols **↔, ⇼** represents bidirectional relationship and no significant relationship, respectively. Also, the symbols **←** and **→** represents one way causation and the arrow direction shows the direction of the causation.

Source: Authors’ calculation based on data from the world bank, UNWTO, and WorldData.info.

**S1.3 Appendix. Granger Causality Test Results – European Region**

| **Regions** | ***ln(PGDP) to ln(TOUR)*** | ***ln(TOUR) to ln(PGDP****)* | **Summary**  ***PGDP - TOUR*** | ***ln(PGDP) to ln(TRADE)*** | ***ln(TRADE) to ln(PGDP****)* | **Summary**  ***PGDP - TRADE*** | ***ln(PGDP) to ln(GCF)*** | ***ln(GCF) to ln(PGDP)*** | **Summary**  ***PGDP - GCF*** |
| --- | --- | --- | --- | --- | --- | --- | --- | --- | --- |
| Europe | 3.1651*** | 2.0331** | **↔** | 1.3872 | 3.5646*** | **←** | 2.9404*** | 11.0240*** | **↔** |
| Albania | 0.1132 | -0.5648 | **⇼** | 1.1197 | 0.0715 | **⇼** | -0.3113 | 2.5944*** | **←** |
| Armenia | 2.1665** | -0.6634 | **→** | -0.6896 | 0.1958 | **⇼** | -0.7062 | 3.3312*** | **←** |
| Austria | -0.3769 | -0.2322 | **⇼** | 0.0241 | -0.6544 | **⇼** | -0.7059 | -0.6992 | **⇼** |
| Azerbaijan | 1.0583 | 0.8657 | **⇼** | 2.4602** | 13.4327*** | **↔** | 1.3344 | 0.0902 | **⇼** |
| Belarus | -0.5128 | -0.0682 | **⇼** | 0.6256 | -0.4018 | **⇼** | -0.3498 | 3.0496*** | **←** |
| Belgium | -0.4976 | 0.1532 | **⇼** | -0.3350 | -0.6444 | **⇼** | -0.5462 | -0.4436 | **⇼** |
| Bulgaria | -0.6870 | -0.4788 | **⇼** | -0.0996 | -0.6709 | **⇼** | -0.6998 | 5.5096*** | **←** |
| Croatia | -0.6476 | 0.1577 | **⇼** | -0.5296 | -0.1981 | **⇼** | 0.3062 | 1.0772 | **⇼** |
| Cyprus | -0.6695 | -0.5430 | **⇼** | -0.0576 | -0.6560 | **⇼** | 6.3193*** | 3.4696*** | **↔** |
| Czech Republic | -0.6842 | 6.4790 *** | **←** | -0.5528 | 0.1638 | **⇼** | -0.3571 | 2.9290*** | **←** |
| Denmark | -0.6927 | -0.6972 | **⇼** | -0.3592 | -0.5471 | **⇼** | -0.6597 | 1.1482 | **⇼** |
| Estonia | -0.5752 | -0.0691 | **⇼** | 0.7695 | 0.2035 | **⇼** | -0.0412 | 2.1859* | **←** |
| Finland | -0.2173 | -0.4297 | **⇼** | -0.6471 | -0.5925 | **⇼** | -0.5629 | 0.4598 | **⇼** |
| France | -0.7066 | -0.0430 | **⇼** | -0.4581 | -0.2874 | **⇼** | -0.5038 | 0.5072 | **⇼** |
| Georgia | 1.4366 | -0.6724 | **⇼** | 3.4651*** | -0.6703 | **→** | -0.7062 | -0.6932 | **⇼** |
| Germany | 5.6702 *** | -0.7064 | **→** | 2.5944*** | 1.1414 | **→** | 15.6920 *** | 1.9915* | **↔** |
| Greece | -0.5437 | -0.2529 | **⇼** | -0.1541 | -0.3562 | **⇼** | -0.7071 | -0.3541 | **⇼** |
| Hungary | 0.1249 | -0.1133 | **⇼** | -0.6460 | 0.4489 | **⇼** | -0.5559 | -0.6117 | **⇼** |
| Iceland | -0.6779 | 1.6784* | **←** | 0.1893 | 0.4975 | **⇼** | 2.8027*** | 0.3926 | **→** |
| Ireland | 0.6289 | -0.7060 | **⇼** | -0.6956 | 13.4483 | **⇼** | -0.6328 | -0.2948 | **⇼** |
| Israel | 0.5696 | -0.5559 | **⇼** | -0.2423 | -0.0626 | **⇼** | 3.4774*** | 3.7279*** | **↔** |
| Italy | -0.2356 | 0.9087 | **⇼** | -0.0160 | 3.4332*** | **←** | -0.5462 | 0.6245 | **⇼** |
| Kazakhstan | 6.5332 *** | 0.7859 | **→** | 1.3838 | -0.4441 | **⇼** | -0.6217 | 7.5229*** | **←** |
| Latvia | -0.7035 | -0.7007 | **⇼** | 0.0813 | -0.6545 | **⇼** | -0.7064 | 4.0987 *** | **←** |
| Lithuania | 0.0299 | 0.1615 | **⇼** | -0.4447 | -0.1492 | **⇼** | 0.6402 | 5.2525*** | **←** |
| Luxembourg | -0.5226 | -0.4051 | **⇼** | -0.3321 | -0.5616 | **⇼** | -0.5279 | -0.6573 | **⇼** |
| Malta | -0.1991 | -0.6077 | **⇼** | -0.7047 | -0.6439 | **⇼** | 0.9797 | -0.6466 | **⇼** |
| Netherlands | 1.0324 | -0.7067 | **⇼** | 0.2600 | -0.4231 | **⇼** | -0.5805 | 0.5068 | **⇼** |
| North Macedonia | 2.3060 ** | 0.7803 | **→** | -0.5068 | 0.2020 | **⇼** | 0.5105 | -0.2546 | **⇼** |
| Norway | 0.8483 | -0.6849 | **⇼** | 0.0942 | -0.6371 | **⇼** | -0.6764 | 1.9152* | **←** |
| Poland | -0.7071 | -0.4916 | **⇼** | -0.7067 | -0.4040 | **⇼** | -0.2885 | 1.1175 | **⇼** |
| Portugal | 2.7649 *** | -0.7015 | **→** | -0.3470 | -0.6992 | **⇼** | -0.1927 | 0.0550 | **⇼** |
| Romania | 2.9309 *** | 2.3798* | **↔** | -0.0072 | 2.9076** | **←** | -0.5919 | 0.8955 | **⇼** |
| Russian Federation | 1.7651* | 0.4025 | **⇼** | 0.3128 | -0.3886 | **⇼** | -0.5696 | 0.8638 | **⇼** |
| Serbia | -0.7070 | -0.6970 | **⇼** | 0.3085 | 0.6317 | **⇼** | -0.4646 | 8.4973*** | **←** |
| Slovenia | 1.4336 | -0.6706 | **⇼** | 0.0818 | 0.1612 | **⇼** | 2.7138*** | 8.2023 *** | **↔** |
| Spain | -0.6087 | -0.4520 | **⇼** | -0.6000 | -0.4891 | **⇼** | -0.1083 | -0.6319 | **⇼** |
| Sweden | -0.3167 | 0.0398 | **⇼** | 2.3254** | -0.4127 | **→** | -0.7070 | -0.6544 | **⇼** |
| Switzerland | 2.0431** | 5.3346*** | **↔** | 2.9579** | -0.5911 | **→** | -0.4157 | 0.2159 | **⇼** |
| Ukraine | -0.3444 | 6.2975 *** | **←** | -0.2139 | -0.5155 | **⇼** | 0.2328 | 2.3531* | **←** |
| United Kingdom | -0.4323 | 0.3635 | **⇼** | -0.7071 | -0.5341 | **⇼** | -0.5434 | 0.0274 | **⇼** |

Note: The symbols *, **, and *** represents 10%, 5%, and 1% significance level, respectively. The symbols **↔, ⇼** represents bidirectional relationship and no significant relationship, respectively. Also, the symbols **←** and **→** represents one way causation and the arrow direction shows the direction of the causation.

Source: Authors’ calculation based on data from the world bank, UNWTO, and WorldData.info.

**S1.4 Appendix. Granger Causality Test Results -** **Asian and Pacific Region**

| **Regions** | **ln*(PGDP)* to**  **ln*(TOUR)*** | **ln*(TOUR)* to ln*(PGDP)*** | **Summary**  ***PGDP - TOUR*** | **ln*(PGDP)* to ln*(TRADE)*** | **ln*(TRADE* to ln*(PGDP)*** | **Summary**  ***PGDP - TRADE*** | **ln*(PGDP)***  **to ln*(GCF)*** | **ln*(GCF)***  **to ln*(PGDP)*** | **Summary**  ***PGDP - GCF*** |
| --- | --- | --- | --- | --- | --- | --- | --- | --- | --- |
| Asia & Pacific | 2.9006*** | 3.0982*** | **↔** | 2.0690** | 14.4490*** | **↔** | 3.5075*** | 4.6961*** | **↔** |
| Australia | -0.7071 | -0.3277 | **⇼** | -0.6168 | 2.4063** | **←** | -0.6985 | 1.4820 | **⇼** |
| Bangladesh | 1.4008 | 1.5864 | **⇼** | 1.2198 | 1.6232 | **⇼** | -0.5857 | 2.9892*** | **←** |
| Bhutan | 5.1330*** | -0.4029 | **→** | -0.0634 | 17.4462*** | **←** | -0.6400 | -0.6556 | **⇼** |
| Cambodia | 1.2399 | -0.5898 | **⇼** | -0.1899 | 0.3242 | **⇼** | -0.5741 | 2.4999** | **←** |
| China | 1.9848** | 4.4959*** | **↔** | 1.4053 | 12.9641*** | **←** | -0.4885 | -0.6910 | **⇼** |
| Fiji | 0.0939 | -0.4801 | **⇼** | -0.3371 | 3.2977*** | **←** | -0.2802 | -0.3510 | **⇼** |
| Hong Kong | 2.7406*** | 7.9995*** | **↔** | 0.9016 | 1.2285 | **⇼** | 5.3621*** | 0.4218 | **→** |
| India | -0.6416 | 0.8391 | **⇼** | -0.7071 | 1.9799** | **←** | -0.6524 | 16.8926*** | **←** |
| Indonesia | 1.0310 | 0.9529 | **⇼** | -0.4072 | 1.9592* | **←** | -0.6702 | 3.2414*** | **←** |
| Japan | -0.5804 | -0.5588 | **⇼** | -0.4415 | -0.5378 | **⇼** | 3.6429*** | -0.6545 | **→** |
| Korea, Rep | -0.1217 | 0.1581 | **⇼** | -0.6268 | 0.1706 | **⇼** | 3.0359*** | 0.3321 | **→** |
| Macao SAR | -0.6475 | -0.7059 | **⇼** | -0.5400 | 0.2124 | **⇼** | -0.7049 | -0.2630 | **⇼** |
| Malaysia | 0.6108 | 0.0660 | **⇼** | -0.7044 | 14.7497*** | **←** | -0.6876 | -0.7044 | **⇼** |
| Mongolia | -0.6693 | -0.6541 | **⇼** | -0.5977 | 0.6028 | **⇼** | -0.6411 | 0.2254 | **⇼** |
| Nepal | -0.5433 | 0.5162 | **⇼** | -0.5210 | -0.6347 | **⇼** | 1.3576 | 0.1380 | **⇼** |
| New Zealand | -0.4885 | -0.1704 | **⇼** | 1.4173 | 1.3430 | **⇼** | 2.7636*** | -0.6900 | **→** |
| Pakistan | -0.6963 | -0.0835 | **⇼** | -0.5270 | 0.3756 | **⇼** | 1.3528 | 0.0510 | **⇼** |
| Philippines | -0.5380 | 0.0060 | **⇼** | -0.5379 | -0.1385 | **⇼** | 0.9688 | 0.2550 | **⇼** |
| Singapore | -0.3850 | 1.1193 | **⇼** | 0.9464 | 4.1379*** | **←** | -0.6800 | -0.7071 | **⇼** |
| Solomon Islands | 1.4890 | -0.5679 | **⇼** | 2.0818** | 0.9080 | **→** | 3.8256*** | -0.6245 | **→** |
| Sri Lanka | 4.9471*** | 0.7437 | **→** | 8.7815*** | 0.8467 | **→** | -0.6295 | -0.7007 | **⇼** |
| Thailand | -0.7070 | -0.6577 | **⇼** | -0.5546 | 3.6967*** | **←** | 0.6481 | 1.3447 | **⇼** |
| Tonga | -0.6694 | 2.4907** | **←** | 0.9404 | 2.7336*** | **←** | 2.2357** | -0.7068 | **→** |
| Vanuatu | -0.6787 | -0.6814 | **⇼** | -0.4723 | -0.2595 | **⇼** | 0.4333 | -0.5940 | **⇼** |
| Vietnam | 1.9059* | 0.3977 | **→** | 0.4959 | 0.8096 | **⇼** | -0.1563 | 0.9502 | **⇼** |

Note: The symbols *, **, and *** represents 10%, 5%, and 1% significance level, respectively. The symbols **↔, ⇼** represents bidirectional relationship and no significant relationship, respectively. Also, the symbols **←** and **→** represents one way causation and the arrow direction shows the direction of the causation.

Source: Authors’ calculation based on data from the world bank, UNWTO, and WorldData.info.
